# Afghan mental health and psychosocial well-being: thematic review of four decades of research and interventions

**DOI:** 10.1192/bjo.2023.502

**Published:** 2023-07-10

**Authors:** Qais Alemi, Catherine Panter-Brick, Spozhmay Oriya, Mariam Ahmady, Abdul Qawi Alimi, Hafizullah Faiz, Nadia Hakim, Sayed A. Sami Hashemi, Muhammad Amin Manaly, Roman Naseri, Khesraw Parwiz, Sayed Javid Sadat, Mohammad Zahid Sharifi, Zalmai Shinwari, Sayed Jafar Ahmadi, Rohullah Amin, Sayed Azimi, Atal Hewad, Zeinab Musavi, Abdul Majeed Siddiqi, Martha Bragin, Wataru Kashino, Michalis Lavdas, Kenneth E. Miller, Inge Missmahl, Patricia A. Omidian, Jean-Francois Trani, Sarah Kate van der Walt, Derrick Silove, Peter Ventevogel

**Affiliations:** School of Behavioral Health, Loma Linda University, California, USA; Jackson Institute for Global Affairs and Department of Anthropology, Yale University, Connecticut, USA; School of Education, Simon Fraser University, Canada; Department of Counselling, Faculty of Psychology and Educational Sciences, Kabul University, Afghanistan; World Health Organization, Kabul, Afghanistan; Jalalabad Regional Management Office, Swedish Committee for Afghanistan, Jalalabad, Afghanistan; Migration Health Unit, International Organization for Migration, Kabul, Afghanistan; Child Protection Section, UNICEF, Kabul, Afghanistan; Action Against Hunger, Kabul, Afghanistan; Mental Health and Psychosocial Support Unit, International Medical Corps, Kabul, Afghanistan; Independent Public Health Expert, Kabul, Afghanistan; Mental Health and Peacebuilding Program, International Assistance Mission, Herat, Afghanistan; Kabul Mental Health Hospital Support Project, HealthNet TPO, Kabul, Afghanistan; Mental Health and Psychosocial Support Unit, HealthNet TPO, Kabul, Afghanistan; Department of Psychology, Bard College, New York, USA; Faculty of Humanities and Social Sciences, Helmut-Schmidt University, Germany; Independent Mental Health Specialist, Geneva, Switzerland; Department of Ipso Academy and Quality Management, International Psychosocial Organisation, Konstanz, Germany; Behrawan Research and Psychology Services Organization, Kabul, Afghanistan; HealthNet TPO, Amsterdam, The Netherlands; Silberman School of Social Work, The City University of New York, New York, USA; Prevention Treatment and Rehabilitation Section, United Nations Office on Drugs and Crime, Vienna, Austria; Department of Psychosocial Science, Faculty of Psychology, University of Bergen, Norway; Faculty of Education, University of British Columbia, Canada; International Psychosocial Organisation, Konstanz, Germany; Focusing Initiatives International, Corvallis, Oregon, USA; Brown School, Washington University in St Louis, Missouri, USA; Mental Health and Psychosocial Support Unit, Première Urgence – Aide Médicale Internationale, Kabul, Afghanistan; School of Psychiatry, University of New South Wales, Australia; Public Health Section, United Nations High Commissioner for Refugees, Geneva, Switzerland

**Keywords:** Transcultural psychiatry, epidemiology, refugees, Afghanistan, conflict and war

## Abstract

**Background:**

Four decades of war, political upheaval, economic deprivation and forced displacement have profoundly affected both in-country and refugee Afghan populations.

**Aims:**

We reviewed literature on mental health and psychosocial well-being, to assess the current evidence and describe mental healthcare systems, including government programmes and community-based interventions.

**Method:**

In 2022, we conducted a systematic search in Google Scholar, PTSDpubs, PubMed and PsycINFO, and a hand search of grey literature (*N* = 214 papers). We identified the main factors driving the epidemiology of mental health problems, culturally salient understandings of psychological distress, coping strategies and help-seeking behaviours, and interventions for mental health and psychosocial support.

**Results:**

Mental health problems and psychological distress show higher risks for women, ethnic minorities, people with disabilities and youth. Issues of suicidality and drug use are emerging problems that are understudied. Afghans use specific vocabulary to convey psychological distress, drawing on culturally relevant concepts of body–mind relationships. Coping strategies are largely embedded in one's faith and family. Over the past two decades, concerted efforts were made to integrate mental health into the nation's healthcare system, train cadres of psychosocial counsellors, and develop community-based psychosocial initiatives with the help of non-governmental organisations. A small but growing body of research is emerging around psychological interventions adapted to Afghan contexts and culture.

**Conclusions:**

We make four recommendations to promote health equity and sustainable systems of care. Interventions must build cultural relevance, invest in community-based psychosocial support and evidence-based psychological interventions, maintain core mental health services at logical points of access and foster integrated systems of care.

The number of countries affected by armed conflict and forced displacement is expected to rise over the next decade, resulting in challenges to human development and socioeconomic integration.^1,2^ Key concerns pertain to the mental health of conflict-affected populations,^3^ systems of psychiatric care and the effectiveness of interventions to support psychosocial well-being. This article presents a synthesis of extant literature on mental health issues, systems of care and culturally relevant interventions for conflict-affected Afghans. It is authored by 30 scholars, practitioners and programme managers, mostly Afghan nationals, who led work on Afghan mental health and psychosocial support (MHPSS). We sought to document significant accomplishments to date, and provide a comprehensive overview to support MHPSS initiatives relevant to Afghans and conflict-affected people globally.

## Afghanistan: sociopolitical developments in the last 40 years

Strategically located at the crossroads of empires in India, Persia and Russia, Afghanistan has a long and turbulent history of war.^4^ In 1979, Soviet forces invaded the country to support the fragile communist regime that had taken control of Afghanistan.^5^ There followed intense resistance from Afghans who rejected attempts to ‘Sovietise’ the country through modernising efforts that clashed with Afghan traditions, encroached upon family decision-making and expanded public roles for women in society.^6^ Deep divisions between Afghan communities and their ruling elites sparked a 10-year war (1979–1989) that was fuelled by massive financial support and supply of arms from foreign powers. Over 6 million Afghans became displaced, the vast majority of whom fled to neighbouring Iran and Pakistan.^7^ Following Soviet withdrawal in 1989, civil war ensued between rival ‘*mujahideen*’ factions, leading to the destruction of much of the capital city of Kabul. This conflict was quelled by the rise of the Taliban, an Islamic fundamentalist group originating among the Pashtun, the largest ethnic group in the country, which promised stability and rule of law.^8^ The Taliban maintained control of 90% of the country from 1996 until they were ousted in 2001 by an international military intervention led by the USA.

After 20 years of Western-backed reconstruction and increasing insurgence, the international forces withdrew in 2021, followed by a rapid takeover of the country by the Taliban and the abrupt termination of the Afghan Government in August.^9^ Just before the 2021 Taliban takeover, Afghanistan's Human Development Index was 0.51, positioning the country as 169th out of 189 nations worldwide in terms of life expectancy, education and overall living standards.^10^ Since then, the situation of women and girls, as well as religious and other minorities, has worsened. Moreover, the economy has suffered dramatically as a result of disrupted markets and the freezing of central bank reserves and loans, pushing millions into extreme poverty and leaving an estimated 55% of the population in need of immediate humanitarian assistance.^11,12^ Many healthcare accomplishments are now in peril, given the regime change in 2021 and the withdrawal of international assistance.^13^

## Afghanistan: Documenting mental health initiatives and research

The past 20 years saw ground-breaking initiatives to establish systems of care to address mental health needs in Afghan populations. Notably, a public mental healthcare system was developed, in-country, to train new cadres of mental health professionals and psychological counsellors and to integrate mental health within the nation's basic healthcare system; such integrated structures of care for mental health are exceptional for a low-income country.^14–16^ Several initiatives were also developed to establish culturally relevant mental health services for Afghans taking refuge in host countries. This paper provides a synthesis of the evidence on the main individual, cultural and structural factors pertaining to Afghan mental healthcare and psychosocial support. It identifies the main factors driving the epidemiology of poor mental health, culturally salient understandings of psychological distress, coping strategies and health-seeking behaviours, and interventions for better systems of mental healthcare. This allows us to reflect upon the daunting task of addressing mental health needs for Afghans, in ways that promote health, dignity and resilience, and ensure equity and sustainability within systems of care.

## Method

### Search strategy

Our review methodology was guided by the ‘Toolkit for Assessing Mental Health and Psychosocial Needs in Humanitarian Settings’.^17,18^ In 2022 (January to July), we searched both peer-reviewed and grey literature sources that focused on Afghan mental health, published from 1978 onward. We systematically searched for papers through Google Scholar, PTSDpubs (formerly PILOTS), PubMed and PsycINFO applying combinations of the following search terms (optimising the ‘AND’, ‘NOT’, ‘OR’ Boolean operators): ‘Afghan*’, ‘mental’, ‘health’, ‘psycho*’, ‘depression’, ‘anxiety’, ‘trauma’, ‘PTSD’, ‘help-seeking’, ‘support’, ‘refuge*’, ‘asylum’. The term ‘Afghan’ had to appear in titles, abstracts or key words as a primary condition of eligibility. Grey literature included unpublished reports produced by the Afghan government and non-governmental organisations (NGOs) providing MHPSS services in Afghanistan, requested through email correspondence.

### Thematic analysis

Two co-authors (Q.A. and P.V.) independently vetted all papers for inclusion, based on titles and abstracts. They then categorised papers with respect to methodology (e.g. historical, correlational, intervention and qualitative studies), sample and setting, measures and outcomes, study purpose and thematic content. The core themes structuring this paper were established inductively, through the careful grouping of studies based on their thematic content (e.g. child and adolescent mental health, suicide, substance misuse). Emerging lack of consensus, with respect to thematic structure, was resolved through consultation and email discussion with all other authors; for example, the author group discussed whether papers on community-based psychosocial programming were best grouped separately from, or together with, papers on psychiatric services and psychological treatment.

## Results

Our search yielded 214 papers (*n* = 143 pertaining to Afghanistan, *n* = 71 pertaining to refugees). This included academic publications in peer-reviewed journals (*n* = 154), books (*n* = 16) or dissertations (*n* = 4); and grey literature comprising government documents (*n* = 7), reports by non-governmental organisations (*n* = 20) and publications by inter-government or donor organisations (*n* = 12). We present our results in two sections. The first is an overview of epidemiological and culturally specific data on Afghan mental health. The second focuses on systems of mental healthcare, community-based psychosocial support and psychological interventions.

### Afghan mental health

#### Epidemiological studies and their limitations

Large-scale, epidemiological mental health surveys were only undertaken in Afghanistan after the fall of the Taliban regime in 2001. Earlier studies had nonetheless highlighted important mental health issues. Thus, in the 1990s, Afghan mental health specialists had raised concern regarding war-related mental health needs.^19,20^ In 2000, women who resided in Taliban-controlled areas were found significantly more likely to report symptoms of depression, suicidal ideation and actual suicidal attempts compared with women residing in non-Taliban-controlled areas.^21^ Symptoms of depression and post-traumatic stress disorder (PTSD) were also high among Afghan women in Kabul and in refugee settings in Pakistan,^22^ which was linked to war-related death and injury of family members, forced displacement and enduring poverty.

Population-based surveys, conducted in 2003, showed that self-reported symptoms of depression, anxiety, PTSD and poor social functioning were very common, particularly among women.^23–25^ Gender-related vulnerability was also reported among those who used primary care services.^26–29^ In Eastern Afghanistan, a 2022 community-based survey identified a highly distressed population, with 53% of respondents indicating they often felt so hopeless that they did not want to carry on living, and 64% indicating they felt so angry that they often felt out of control.^30^ Among Kabul University students in 2021, in the aftermath of the fall of the Ashraf Ghani Government, 70% of respondents reported significant symptoms of PSTD and/or depression, and 39% indicated heightened suicide ideation/behaviour.^31^

These studies were based on self-reports with questionnaires that were not validated for culture and context. This raised questions regarding the validity of thresholds used to demarcate psychiatric disorders from psychological distress, and just as importantly, questions regarding the face validity and relevance of psychiatric symptoms and trauma reports for Afghan populations.^32^ Subsequent clinical validation of a widely used screening tool, the Hopkins Symptoms Checklist-25, in Afghanistan and among Afghan refugees in Japan found only moderate agreement between the results of screening and clinical interviews.^33,34^

The above studies must, therefore, be read with a great deal of caution: high prevalence estimates likely conflate mental disorders with psychosocial distress,^35^ whereas culturally grounded indicators may be more suited to assess severe mental distress.^36^ Indeed, the 2018 National Mental Health Survey, commissioned by the Afghan Government, found markedly lower prevalence estimates for adults (*N* = 4445), using a diagnostic standardised interview (the Composite International Diagnostic Interview Short Form) rather than self-report screening questionnaires. In this probability survey by multistage sampling, 12-month prevalence of PTSD was 5.3%, major depressive episode was 11.7% and generalised anxiety disorder was 2.8%.^37^

#### Adult mental health

Studies in Afghanistan have documented^.^ high levels of exposure to potentially traumatic events. For example, in the 2003 study by Scholte et al, 57% of adults surveyed had experienced more than eight potentially traumatic events over the past decade, including shortages of food, lack of access to medical treatment and lack of shelter.^25^ However, the associations between trauma events and mental health symptoms are not always straightforward. For example, the 2003 study by Cardozo et al did not find significant associations between trauma exposure and PTSD symptom criteria, only with anxiety; this was attributed to extreme economic hardship elevating stress and anxiety, without associated PSTD.^23^ The point is important, and reiterated in many studies: among Afghans, mental health issues do not arise only from war-related violence. They arise from everyday stressors related to human insecurity, in the wake of poverty, forced displacement, the disruption of family and community support, and the loss of housing and livelihoods.^16,38–41^

Gender differences in depression and anxiety are clearly pronounced in Afghanistan.^38,42,43^ These are attributed to the systematic and institutional discrimination experienced by Afghan women in many parts of the country^44^ including the system of *purdah*, a practice of strict gender segregation excluding women from public spaces.^45^ Gender difference in mental health issues may also be related to the cultural ‘straitjacket’ governing the public expression of emotions, discouraging women to reveal forms of despair and men to show fear, grief or doubt, as this would bring shame on the family.^35,46,47^

For young adults, studies have linked common symptoms of distress with higher education, ethnicity, income instability and reduced levels of hope and optimism with regard to one's own trajectory and the country's future.^48^ This may reflect a pervasive disillusionment with a national economy that cannot provide job opportunities for many university graduates. Indeed, a survey of a representative sample of Kabul University students in 2008 demonstrated that both men and women were frustrated by the conditions that led to widespread insecurity and prevented social advancement.^49^ Using biomarkers to measure individual-level stress (*N* = 161), this study confirmed the salience of family stressors for women's mental health and significant associations between psychological and physiological markers of well-being. Biomarker stress data demonstrated that, for Afghans, the gendered structure of everyday life had consequences for both stress physiology and mental health.

#### Child and adolescent mental health

Among children and youth, community-based surveys and ethnographic research have shown that family-level violence, more so than war-related violence, is an important driver of mental health problems such as depression and anxiety.^50–52^ In 2006–2007, a prospective study in Afghanistan and among refugees in Pakistan by Panter-Brick et al used stratified random sampling to survey adolescents, parents and teachers (*N* = 3014) to assess mental health, traumatic experiences and social functioning. It found that both war-related and family-level violence had demonstrable effects on the mental health of 11- to 16-year-old children.^42^ It also demonstrated strong associations between caregiver and child mental health: 1 s.d. change in caregiver mental health was associated, at follow-up, with a 1.04-point change on child post-traumatic stress symptoms, equivalent to the predictive impact of a child's lifetime exposure to one or two trauma events, as well as a 0.65-point change in depressive symptoms, which was equivalent to two-thirds of the effect attributed to female gender.^53^ The study also documented that trauma memories were malleable over time, presenting heterogeneous associations with post-traumatic distress. Because traumatic events were embedded in social experiences, being shaped by family and cultural narratives of suffering and resilience, trauma was best described as both a clinical and a social event. Cases of domestic violence were a case in point, being reported as trauma only when described as ‘senseless’ – beyond normative disciplinary violence, or stressful outbursts of violence triggered by insecure employment, housing or school difficulties.^42,51^

Again, this cautions against simplifying assumptions linking trauma, war-related violence and mental health that elude consideration of family functioning or socioeconomic factors. For example, a 2013 survey of displaced 15- to 24-year-old youth (*N* = 2006) in Kabul reported that both everyday and militarised violence affected mental health,^54^ and 2016 interviews with 10- to 21-year-olds in Kabul, Kunduz and Balkh showed that violence had become a ‘normalised’ part of daily life-worlds, shaping almost all social interactions.^52^ Harsh discipline is often related to a parent's own experience of family-level violence, as found in a study of women who stated witnessing their own mothers being physically abused and mentioning how they acted violently toward children when feeling distressed.^55,56^ Even in schools, child-on-child violence has been linked to exposure to home-based violence.^57^

Data from after the fall of the Ghani Government, although scarce, point to a deterioration of the psychosocial well-being of children. A 2021 study of 10- to 12-year-olds in rural primary schools of Badakhshan, Ghazni and Takhar provinces, using culturally adapted measures of depression and anxiety, found that 52% of children suffered from depression, including 2.6% from severe forms. Almost all children showed some signs of anxiety, 23% severely so. In a sample of 376 high school children in Kabul, approximately half met the criteria for a probable diagnosis of PTSD (*n* = 194, 51.6%), depression (*n* = 184, 48.9%) or anxiety (*n* = 170, 45.2%).^58^

#### Gender-based violence

Gender-based violence is a known issue, but one difficult to report and address given the salience of family patriarchy and fear for social exclusion.^59^ Afghanistan ranks highest among low- and middle-income countries in terms of past 12-month prevalence of physical, emotional and sexual violence.^60^ Population-based surveys have found that close to half of Afghan women reported exposure to violence from their husbands.^61,62^ Globally, Afghanistan is rated the worst place to be a woman or a girl.^63^

Although the literature linking gender-based violence and mental health is sparse, a recent population-based study argued that Afghan women carry the double burden of war-related and gender-based violence, leading to high lifetime suicidal attempts and risks of depression and anxiety.^37^ In Eastern Afghanistan, for example, key informants indicated massive distress among women, because of violence in their homes.^30^ Research with perpetrators of gender-based violence in Afghanistan is extremely rare. In a national survey, Afghan men indicated that wife-beating is acceptable, whereas overt aggression over family members is not, and that aggressive behaviour is enacted out of frustration when unable to fulfil societal expectations.^64^ Mental health professionals working in Afghanistan have also argued that men use violence in the home to counter their feelings of powerlessness.^16^

#### Suicide

A systematic review on seven studies on suicide and self-harm, based on hospital admissions, has drawn attention to women's markedly elevated rates of suicidality and self-harm, mostly in the form of self-immolation.^65^ In Afghanistan's 2017 National Mental Health Survey (*N* = 4474), 2.2% of adults reported suicidal thoughts in the past 12 months and 3.4% reported that they had, at least once in their life, attempted suicide, with women almost doubly at risk compared with men.^66^ Within samples of women with clinical mental health issues, suicidality is particularly high: a study that randomly sampled 117 patients in a mental health clinic in Herat, comprising mostly women with depression, showed that 78% had reported suicidal ideation and 30% had thought about poison and self-immolation.^67^ An analysis of 77 cases of self-immolation showed that half of the victims were young (16–19 years old); four out of five had died.^68^ As identified by relatives or survivors of self-immolation, precipitating events included a forced marriage (29%), a practice known as ‘*bad*’ in which girls are exchanged as brides to settle family or clan conflict (18%), or abuse from in-laws (16%). Most scholars relate the drivers of self-harm to household conflict and forms of violence against women.^69,70^ Some observers have argued that self-harm may best be perceived as a form of social protest against oppression, often related to arranged and forced marriages.^71^

#### Substance use

Data from a drug use survey by the United Nations Office on Drugs and Crime in Afghanistan have suggested exponential increases in heroin use among Afghan adults between 2005 and 2009,^72,73^ leading to adult drug use rates that are more than twice the global average.^74^ Among 13- to 18-year-olds, a drug use survey in 2019 showed considerable use of illegal substances among secondary school students (14% for boys, 8.5% for girls). A new problem within Afghanistan is the use of methamphetamine: 1.3% of Afghan students reported that during the past year they had used methamphetamine in powder form or as tablets known as ‘tablet K’.^75,76^

In 2021, a survey by the International Medical Corps across northern and eastern regions of Afghanistan showed that men endorsed the use of narcotic drugs as a serious problem, and that community leaders identified addiction as linked to mental health issues.^77^ For its part, the Afghanistan National Urban Drug Use Study, which relied on analyses of hair, urine and saliva samples from 5236 people in 2187 randomly selected households, showed that one in 20 (5.6%) biological samples showed evidence of opioid use. From laboratory tests, the estimated prevalence of substance use was 7.2% in men and 3.1% in women.^78^

Research on substance use is fraught with challenges. Studies have indicated that drug use is widespread for refugees displaced to Iran and Pakistan,^79^ linked to feelings of loss, distress and boredom in Iran, and limited economic mobility in Pakistan.^80^ Afghans with substance use problems reported being widely stigmatised in Iran.^81,82^ There are reports of Afghan women, struggling to balance livelihoods and childcare responsibilities in Pakistan, giving opium to children to keep them tranquil.^80,83^ For women, access to substance use services has been very low;^74^ heroin use often leads them to being disowned by their families because of the shame associated with addiction.^84^ Detoxification and addiction treatment programmes are underutilised among those who use injection drugs.^85,86^

#### Afghan refugees

As expected, studies find high PTSD and depression symptoms among Afghans displaced to countries such as Iran,^87^ Pakistan,^88,89^ Turkey,^90^ Serbia^91^ and high-income countries such as Australia,^92,93^ Canada,^94^ Germany,^95^ The Netherlands^96,97^ and the USA.^98^ Poor mental health lingers for long periods after resettlement,^99^ given that the associations between trauma exposure and clinical symptoms are moderated through the presence of ongoing stressors and loss of coping resources.^100^ Post-resettlement stressors include a state of precarity with regards to residence status,^101,102^ loneliness,^93,103^ acculturation difficulties,^104^ unemployment, intergenerational conflicts,^59^ gender role changes,^105,106^ discrimination,^107^ education and inclusion.^108,109^

Notably, in some settings, Afghan refugees and asylum seekers face important restrictions on their mobility, with migration detention having both short-term and long-term adverse psychological effects.^110,111^ For example, Afghans confined to camps on the Greek islands reported high levels of psychological stress, related to their powerlessness and pessimism regarding current and future prospects.^112,113^ Similarly, Afghan refugees and asylum seekers in Serbia and Norway have reported high levels of desperation and frustration, in the context of de-humanising experiences at the hands of legal systems.^91^ Asylum procedures aggravate mental health conditions and recovery; this includes the experience of Afghan adults and children taking refuge in high-income countries.^102,114,115^ For example, a study among unaccompanied asylum-seeking Afghan children in the UK found that PTSD symptoms were associated with pre-migration traumatic events (cumulative stress) and living arrangement (foster care versus independent living).^116^ In Sweden, unaccompanied refugee minors had a higher prevalence of PTSD compared with Syrian and Iraqi children.^117^

#### Culture and mental health

Our search returned 13 papers describing how Afghans understand different forms of mental health behaviour, psychological distress, well-being and resilience (Table 1). For example, De Berry et al's qualitative work in Kabul explored concepts of psychosocial well-being, providing a range of local idioms that subsequently informed support programmes for war-affected children in Afghanistan.^118^ Miller et al's seminal work provided important insights into what it meant to be ‘doing well’ and ‘doing poorly’ psychologically.^119^ Their study identified several indicators of poor mental health, some culturally specific, others known to Western psychiatry, and used these to construct a mental health screening tool: the Afghan Symptom Checklist. A subsequent study tested the extent to which Western and culturally specific conceptualisations of distress have unique physiological signatures, mapping on systolic and diastolic blood pressure.^124^ In turn, Eggerman and Panter-Brick's mixed-methods study^41^ documented the contexts in which Afghans articulated poor mental health and psychological distress, emphasising how Afghans saw clear links between health, lives and livelihoods: poor mental health was linked to war-related violence and the loss of loved ones, as well as the broken economy, inadequate shelter, poor education opportunities and insecure governance. The study also provided insights into how key cultural values (faith, family unity, service to the community, perseverance, morals and respectability) were the cultural backbone of psychosocial resilience in the face of ongoing adversities.

**Table 1:** Culturally salient understandings and idioms of mental health and psychosocial well-being, as narrated by Afghans

Study purpose (reference)	Sample and setting	Understandings of mental health and idioms of distress
Explore how Afghan children and caregivers define emotional well-being^118^	Children and adults (approximately 600) participating in focus group discussions (*n* = 238) in Kabul	Emotional states range from severe mental illness (*rawany taklef*) and madness (*dewana*) to expressions of emotional distress such as: *gham –* sorrow and depression *tashweesh –* worry *khafaquan* – feeling strangled *tars* – fear These conditions are usually expressed through somatic complaints
Test a methodology for identifying local beliefs and developing culturally grounded measures to assess psychological well-being and distress^119^	Adults from 16 districts in Kabul city (*n* = 162 men; *n* = 162 women)	Three dimensions of psychosocial well-being centred on functioning in the community, the family and one's internal state – related, respectively, to social respect, family harmony and calm or agitation. Culturally salient idioms and expressions of distress included: *Asabi –* nervous agitation *Fishar bala –* emotional pressure and agitation *Fishar payin –* low energy and motivation *Ghamgeen –* sadness *Jigar khun –* grief following interpersonal loss, or reaction to deeply disappointing or painful experiences Quarrelling with family, neighbours or friends Thinking too much
Document views on health, illness and mental worries^41,120^	Adult refugees in The Netherlands (*N* = 36)	Health was evidenced from physical, social, emotional and mental functioning, and from autonomy; mental illness showed as worries or ‘thinking too much’ because of loneliness, unemployment, war experiences, loss of family members, separation from family or inability to support the family
Present a thematic analysis of narratives related to adversity, suffering and resilience^41^	Afghan families in four districts in Afghanistan (*n* = 1011 children; *n* = 1011 caregivers; *n* = 358 teachers) and in refugee camps in Pakistan (317 child–caregiver dyads)	Idioms of psychological distress are rooted in the body, and clearly differentiate between anger, stress, melancholy and anxiety. They include: *Takleef asabi –* irritability and anger *Fishar payin/fishar bala –* lethargy and agitation, as well as blood pressure *Jigar khun –* a state of acute dysphoria, sorrow, regret and depression, often because of losing relatives as a result of war *Tashweesh –* everyday worry (denoted by expressions such as *delam naram hast* or *delam az-zindagi sard shoda)* Mental health, resilience and fortitude rest on a sense of hope for the future: a sense that adversity can be overcome. Hope arises from a sense of moral and social order, embodied in the expression of six key cultural values: *Iman –* faith *Wahdat and ittifaq –* family unity and harmony *Khidmat –* service to parents, family and community through achieving educational and economic aspirations *Koshesh –* perseverance and effort *Akhlaq –* morals *Izzat -* social prominence, respectability and honour
Identify resettlement experiences, mental health outcomes and ongoing sources of stress^121^	Afghan refugees in Australia and New Zealand (*N* = 90)	‘Thinking too much’ was the most commonly mentioned idiom, rooted in past experiences and current reminders of trauma, characterised by feelings of unhappiness, sadness and depression
Explore conceptualisations of depression^122,123^	Afghan refugees in the USA (*N* = 111)	*Afsurdagi* is the Dari term for depression: Causes include pre-migration traumas (witnessing atrocities, losing family because of displacement and death), post-resettlement stressors Symptoms include *asabi* (irritability), *ghamgeen* (feeling sad), *goshagiry* (self-isolation), thinking too much, inability to complete daily tasks and a range of somatic complaints Treatments include exercise as well as rest, religious activities, visits to Afghanistan and help-seeking from mental health professionals and traditional healers
Test association between the idiom of psychosocial pressure (*fishar*) and the physiological metric of blood pressure^124^	Adult men and women in Afghanistan (*N* = 991)	The Afghan idiom of *fishar (*‘psychosocial pressure’) corresponds with physiological blood pressure (systolic and diastolic blood pressure) remarkably well compared with other cultural concepts of distress related to anxiety/nervousness (*asabi*) and grief/dysphoria (*jigar khun*)
Learn about views of psychosocial well-being, expressions, operationalisations and conditions for achieving well-being^125,126^	Afghan adults from four regions of Afghanistan (*N* = 440)	Indicators of psychosocial well-being included: *Aram –* peace of mind *Rahat–-* to be comfortable and relaxed with a free state of mind *Khob –* good *Khosh –* happy Conditions for achieving well-being: Peace, security, justice; love/support in the family, freedom, physical health, economic security, access to resources; participation in cultural and religious practices; friendship/support outside the family; self-efficacy/self-esteem; leisure activities
Illustrate the ongoing adaptation process of culturally adapted CBT with Afghan refugees^127^	Afghan adults residing in Germany (*N* = 24)	Derived cultural concepts of distress from previous research, to include the following idioms: *Asabi –* nervous agitation *Gham –* sadness *Jigar khun –* a general expression of intense psychological distress *Tashweesh –* worry *Goshagiry –* self-isolation *Fekro khial –* rumination and worrying *Faramooshi –* forgetfulness
Investigate explanatory models of depression and PTSD^128^	Afghan adults residing in Norway (*N* = 27)	Based on a vignette of a fictional person with symptoms of depression or PTSD, the causes and risk factors of depression and PTSD were identified as follows: Women emphasised gender issues in both depression and PTSD, referring to forced marriages, gender roles, domestic violence, harassment and violence against women, social control and generational conflicts Men emphasised acculturation challenges, such as isolation and loneliness, and concerns fleeing Afghanistan and seeking asylum For managing the symptoms of depression and PTSD: Women mentioned talking to a trusted person (husband, family member, friend) to clear negative thoughts and feelings; the family was viewed as integral in promoting positive mental health for women Men mentioned the importance of having a job, reconnecting with family and socialising with friends, as well as managing symptoms through religious activities, setting goals and engaging in activities
Explore how young people describe mental health underlying factors	Afghan Hazara refugees in Australia (*N* = 18)	Mental health is viewed as interconnected with physical health and the environment. Poor mental health is described as an ‘illness of thoughts’ manifested through headaches, red eyes, disturbed or excessive sleeping. Young men attributed mental health problems to changes in gender roles in Australia Both men and women endorsed family separation as a cause of mental health problems, especially for unaccompanied minors Mental illness stigma was a concern, discouraging people from talking about their feelings and seeking support

CBT, cognitive–behavioural therapy; PTSD, post-traumatic stress disorder.

Culturally salient expressions of depression include the concept of ‘thinking too much’,^120,121,127^ closely aligning with formal psychiatric classifications pertaining to temperament, cognition and functioning. Somatic complaints are prominent, in the form of headaches, chest pains, tension in the body or pins and needles. For example, ethnic Hazara refugees in Australia described poor mental health as an ‘illness of thoughts’, manifested through headaches, red eyes, disturbed sleeping or sleeping a lot.^125^ Specific Dari and Pashto idioms designate sorrow, pain and suffering, such as *‘gham’*,^122,123^ through which women gain social respectability.^129^ Local explanations of PTSD symptoms have equated these to fear.^128,130,131^ Finally, concepts of well-being, such as *‘aram’* (‘feeling psychologically and socially well’) and *‘rahat’* (‘feeling comfortable and relaxed, with free state of mind’), are closely linked to peace and security, strong family relationships, friendship/support outside family and engagement in religious/cultural practices.^126^

#### Coping

Research on health-seeking behaviours has been limited. Afghans are reluctant to self-identify as having mental health issues,^132^ which puts a major stress upon families.^133^ Afghans with persistent states of sadness may seek help from psychiatrists, ‘*mullahs*’ (religious leaders) or *‘tabibs’* (herbalists); take anti-depressants or turn to religious activities. Thus, in Afghanistan, mentally ill people are traditionally brought to *mullahs*, then to healing centres, often centred around the tomb of a holy man, such as the Sufi shrine of Mia Ali Baba.^134,135^ Early epidemiological research documented that people who feel ‘sad, worried or tense’^25^ relied upon Islam as a source of faith and upon family support. In Kabul, young people sought help from both formal and informal sectors; namely, from primary care physicians, as well as from *mullahs* and *tabibs*.^136^ One study reported that Afghan refugee youth in Iran resorted to imagination and fantasies to cope with exposure to extreme violence.^137^ In Australia, asylum seekers have turned to general practitioners^138,139^ and mental health professionals,^125^ but have also drawn on faith and informal social networks for support.^105,140^ Higher symptom severity was often the prompt for seeking care.^141^ One national survey (*N* = 5130 households) found that persons with disabilities^142^ experienced stigma from birth, because disabilities were attributed to a divine curse or the actions of a *‘jinn’*.^143^

Afghans have often been cited as a people living with singular resilience and fortitude among the ongoing challenges of war and socioeconomic adversity. As national and local structures of support were visibly broken, they came to rely on family and community, the only reliable social sources of support left to them.^41^ One case study illustrates what this means for a widowed mother of three, who experienced domestic violence.^144^ She turned to her husband's first wife and her mother-in-law, then found other women who shared similar hardships; this calmed her feelings and showed she was not alone in her struggles. Webs of informal social support have also been reported for Afghan refugees living in the USA.^145–147^ Most studies have emphasised that such ways of coping can be quickly undermined by social and economic isolation, and that research on mental health risk factors need to be balanced with in-depth understanding on cultural resources, social functioning, psychosocial well-being and resilience.

### Mental healthcare and community-based interventions

#### Mental health services in Afghanistan

The government first initiated community-based mental health services during communist rule: in 1985, a Department of Mental Health was established within the Ministry of Public Health, with four community mental health centres in Kabul. In 1992, these care facilities were looted when the Soviet-backed government fell,^148^ and throughout the civil war and Taliban regime, Afghanistan's rudimentary mental health system was severely disrupted.^134^ A major step forward was taken in 1999, when a 3-month diploma course was initiated to train 20 medical practitioners in basic psychiatric practice;^149^ it could not be sustained, given prevailing insecurity. By 2002, only two psychiatrists and a few dozen doctors were working within mental healthcare facilities, most of them with limited training.^150^

In the early 2000s, the Afghan Government and donor communities made a strategic decision to contract out the reconstruction of Afghanistan's healthcare system to NGOs.^151^ In Nangarhar province, HealthNet TPO, tasked with healthcare reconstruction, took steps to integrate mental health into basic healthcare, focusing on priority conditions such as depression, anxiety, psychosis and epilepsy.^152^ This initiative resulted in significant increases in service utilisation for mental health, from fewer than 0.5% of all consultations in the healthcare system to around 5%.^153^ The programme was later rolled out to another six provinces, where, from 2005 to 2008, over 125 000 consultations related to mental health were registered, mostly for depression (66.6%), anxiety (14.9%), epilepsy (9.5%) and psychosis (4.3%), but also for intellectual disabilities (1.2%), substance use disorders (0.7%) and unexplained somatic and other complaints (2.8%).^154^

A key document in the reconstruction of the Afghan health system was the Basic Package of Health Services (BPHS), published by the Afghan government in 2003.^155^ This document (Table 2) played a pivotal role in the remarkable increase in healthcare coverage in the first decade of reconstruction. It described the minimum services to be made available at primary health centres throughout the country, including community management of mental problems and health facility-based treatment of out-patients and in-patients with mental health conditions.^163^ In 2005, a national coordinator for mental health in primary healthcare was appointed in the Afghanistan's Ministry of Health, subsequently establishing a mental health unit.^164^ Afghanistan's first national mental health strategy was thus explicitly designed to improve access to formal mental health services, moving away from hospital-based care toward integration into the primary care sector.^160^ This led to massive upscaling of efforts to train general health workers in identifying and managing the most prevalent mental health conditions.^15,165–167^ Up until 2018, NGOs trained almost 1000 physicians from all provinces through 12-day trainings in basic mental health, and over 1200 nurses and midwives received a 6-day basic mental health training, with curricula developed by the Afghan Ministry of Public Health.^1^ The ‘mhGAP Intervention Guide’, a clinical tool to assist non-specialist health workers to identify and manage mental health conditions,^168^ was introduced on a smaller scale, in three provinces in 2013. In 2022, the World Health Organization (WHO) conducted a national training of trainers in the WHO's Mental Health Gap Action Programme (mhGAP).^2^

**Table 2:** Milestones in the development of mental health services in Afghanistan

Year	Milestone	Source
1985	Department of Mental Health established in the MoPH, Kabul	^148^
1987	First Mental Health Act ratified by Parliament	^156^
1987	First psychologists and social workers graduate from Kabul University	Personal communication, Dr Sayed Azimi
1988	Kabul Psychiatric Hospital (first hospital dedicated to mental health) established with 100 beds	Personal communication, Dr Sayed Azimi
1988	First mental health training manual (‘Mental Health for All’) for general practitioners, authored by Dr Burna Asefi, then Director of Mental Health at the MoPH	Personal communication, Dr Sayed Azimi
1989	First drug treatment centre in Afghanistan, established at the Khoshhal Khan Polyclinic, with 20 beds, supported by the UNDCP and WHO	Personal communication, Dr Sayed Azimi
1992	Looting of mental health facilities; closing of the mental health department of the MOPH in Kabul	^148^
2003	Basic Package of Health Services introduced, with mental health as one of the seven modules	^155^
2004	Mental health unit in Ministry of Public Health re-established	Personal communication, Dr Sayed Azimi
2005	Treatment manual published (‘Mental Health in Primary Health Care: Diagnosis and Treatment of Priority Mental Health Conditions in Afghanistan's Basic Package of Health Services’)	^157^
2005	Mental health included in the Essential Package of Hospital Services	^158^
2005	Directorate of Drug Demand Reduction established in Ministry of Public Health	Personal communication, Dr Mohammad Raza Stanikzai
2009	Revised version of the Basic Package of Health Services, which includes psychosocial counsellors as staff in comprehensive health centres	^159^
2011	National Mental Health Strategy 2011–2015 launched	^160^
2013	Department of Counseling at Kabul University established	^161^
2016	First group of 35 counselling psychologist graduates at Kabul University	Personal communication, Mariam Ahmady
2017	First National Suicide Prevention Strategy developed	Personal communication, Dr Sayed Azimi
2018	National Mental Health Strategy 2019–2023 launched	^162^
2019	Start of the 2-year diploma training programme for health social counsellors, by the Ghazanfar Institute of Health Science	Personal communication, Dr Majeed Siddiqi

Dr Sayed Azimi is an independent mental health specialist in Switzerland who served in various positions with the Ministry of Public Health and the World Health Organization in Afghanistan. Mariam Ahmady was until autumn 2022 Chairperson of the Department of Counseling at Kabul University in Afghanistan. Dr Mohammad Raza Stanikzai was National Programme Coordinator of Country Office for Afghanistan of the United Nations Office on Drugs and Crime. MoPH, Ministry of Public Health; UNDCP, United Nations International Drugs Control Programme; WHO, World Health Organization.

A major innovation was to appoint psychosocial counsellors as mandatory staff in comprehensive health centres, following the revised BPHS in 2009.^159,169,170^ NGOs like Ipso and HealthNet TPO trained hundreds of men and women in the role of psychosocial counsellors.^14,167^ For example, Ipso established a 1-year training course and developed a standard curriculum for psychosocial counsellors with the Ministry of Public Health: this consisted of 3 months of intensive class-based training, followed by 9 months of practical work under supervision and two refresher training courses lasting 2 weeks. The training of this new cadre of health professionals became an accredited diploma programme of the Ghazanfar Institute of Health Sciences, under the Ministry of Public Health. Thus in 2019, around 750 psychosocial counsellors were embedded within the national health system.^171^ The Ghazanfar Institute also upgraded its curriculum to establish a 2-year diploma for health social counsellors, through which 190 people graduated up until 2021. Programmes in counselling psychology were also introduced into public universities by the Ministry of Higher Education,^161,172,173^ with 170 counselling psychologists (68% female) receiving a Bachelor's degree. Thus, in 2018, a Model Counselling Centre was established at Kabul University, playing a key role in improving students’ counselling and psychotherapeutic skills. Even after the regime change in 2021, the Department of Counseling continued to function, training 199 students (68% women) in gender-segregated classes as of July 2022.^3^

Mental health treatment was also initiated in district hospitals and provincial hospitals. Before 2004, the secondary mental health system in Afghanistan was limited to one 60-bed national mental hospital in Kabul and some psychiatric wards in provincial hospitals.^174^ The inclusion of mental health services in a priority list of interventions for secondary care,^158^ in itself remarkable, led to the establishment of psychiatric wards in the five regional hospitals and in provincial hospitals, despite issues with staff retention and equipment.^4^ The National Mental Health Strategy 2019–2023 strived for integration of mental health services into all 83 district hospitals and 27 provincial hospitals; five regional mental health wards provided in- and out-patient specialised services, including residency programmes for psychiatrists.^162^ In reality, however, most district hospitals and provincial hospitals did not provide mental healthcare.

Integrating mental health in public systems of care Afghanistan required overcoming major financial, human, infrastructural and information resource limitations.^175^ Major challenges pertained to adequate staffing, quality of care and on-the-job supervision, given that clinical supervisors were often unable to visit health centres because of transport and security risks. Population needs were high: from 2017 to 2022, the governmental health information system registered around 2 million mental health conditions in primary care per year, constituting around 4% of the total consultations in primary care.^5^ But despite attempts to improve hospital conditions and strengthen staff capacity,^176^ hospitals remained poorly staffed, with patients held in substandard conditions and treatment largely limited to pharmacotherapy. A 2015 assessment identified serious gaps in service delivery and gross disregard for human rights. Patients were routinely exposed to forced treatment and physical restraint, enduring verbal, physical and emotional abuse.^177^

Despite all efforts, the mental health system in Afghanistan is far below acceptable levels of service provision. The number of psychiatrists increased from just two in 2002 to over 100 in 2019:^171^ as of mid-2019, there was approximately one psychiatrist and one psychologist per half-million population.^178^ Rural dwellers struggle to access services because of insecurity, distance to health facilities, travel costs, medication expenses and/or private doctor fees. Household expenditures for health services are high, and the country does not provide a social insurance scheme.^179^ Poverty, treatment costs, stigmatising beliefs and limited support are main barriers to the care of mental disorders.^180^

With respect to drug use, Afghanistan reportedly had 104 treatment centres, providing residential, out-patient and home-based services for about 30 000 clients annually; this is a small portion of the estimated 2.5 million Afghans with substance use disorder.^181,182^ One evaluation study found that intensive treatment (10 days of detox, with 30–45 days of in-patient treatment and 12 months of out-patient treatment) led to significant reductions in opiates and other drug usage.^183^ Thus use of opioids fell by 39% among 865 people, a year after treatment.^181^ However, such a comprehensive service is exceptional; in most cases, there is insufficient follow-up after detoxification, and many drug treatment centres report high rates of relapse,^6^ especially in the absence of recovery support, such as motivational techniques associated with successful treatment completion.^184^ Opioid agonist treatment, based on ongoing methadone treatment, is rare and politically sensitive, and the opioid agonist treatment centre established by Médecins du Monde^185,186^ has now closed. Since August 2021, 44 drug rehabilitation centres are known to be closed; others operate without sufficient funds for staff salaries and medical supplies. Of the 16 centres in Kabul, only four still provided services in 2022, given the halt in donor funding.^182^

#### Community-based initiatives

Many international organisations launched community-based psychosocial support programmes after the 2001 fall of the Taliban. Such programmes were rooted in socioecological models of well-being,^187–191^ recognising that local cultural practices, traditional resources and social support systems can help preserve psychosocial well-being and resilience; for example, Afghan women have strong traditions of narrative storytelling, which provides opportunities for psychological healing.^192^ Such programmes understood that restoring capacities in families and communities to manage psychosocial issues is often more pressing than launching interventions based on clinical expertise. They also saw the need to develop interventions in close consultation with community stakeholders.^193^ Community-based psychosocial initiatives thus often involved participatory workshops with community workers and school teachers, and where possible, drew upon local coping and healing traditions.^187^ They also engaged with government structures to train Afghan professionals in social work and community development, propelling the Afghan Government, as part of the National Skills Development Program, to create national social work standards and curricula for social work education.^194^

Some of these initiatives built upon earlier work with Afghan refugee communities in Pakistan. For instance, the psychosocial training programmes for Afghan teachers, developed by the International Rescue Committee in Peshawar's refugee camps, was moved into Afghanistan.^195,196^ War Child Holland used ‘community action planning’ in Herat to improve children's well-being through play in safe spaces and intergenerational collaboration.^197^ HealthNet TPO used a ‘community systems strengthening’ approach aimed at empowering people in Afghan villages to restore social cohesion, rebuild community trust and create a responsive and supportive environment for those in need. *Mullahs*, teachers and other community members were considered equal partners in improving psychosocial well-being, and also women's exposure to domestic violence, in culturally relevant ways.^198^

Rigorous evaluations of community-based psychosocial initiatives were hardly ever conducted, with the notable exception of those conducted by a coalition of international NGOs implementing child-focused psychosocial activities in 150 villages in Northern Afghanistan. These activities included the creation of ‘child-centred spaces’, offering non-formal education and the establishment of community-led ‘child well-being committees’, engaging children, adolescents and adults to identify child protection threats and livelihood initiatives.^193,199^ They also aimed to help war-affected children to re-socialise through playing and learning, in the context of peace promotion within Afghan society.^189^ Evaluations showed a positive impact on school attendance, children's feelings of safety and inter-ethnic social interactions,^200^ and reductions in aggressive behaviours were sustained a year later.^201^ One evaluation used a quasi-experimental framework to compare a stand-alone psychosocial intervention with a participatory water-sanitation intervention; interestingly, the latter had larger positive effects on child and adult well-being a year later.^200^

‘Focusing’, a body-based mindfulness practice developed in the USA, was introduced in Afghanistan as a self-healing method outside clinical settings. The intervention aimed at helping people listen to each other and to themselves with kindness and positivity, and to understand how emotional reactions to events are intrinsically related to their body's protective responses. ‘Focusing’ was adapted to Afghan values, taking into account family structures and everyday stressors, using local language to describe the process of mindfulness and drawing from Persian and Afghan poetry.^202^ This approach served to anchor community-based psychosocial support programmes with schoolteachers, vocational training groups and NGO staff in Afghanistan. Thus, from 2002 to 2008, the American Friends Service Committee (AFSC) worked with local partners and Kabul University to give eight student interns per year extensive training in psychosocial support and in ‘focusing’, with classroom learning and supervised field experiences. The 10-h programme was designed to support trauma recovery, resilience and conflict resolution in non-medical settings. Over a 6-year period, the AFSC recorded that 48 trainers delivered the psychosocial support training package to 3242 teachers (AFSC internal reports 2002–2009). External reviews found that the method was easily embraced by both literate and non-literate Afghans.^203,204^ Major donors such as the United States Agency for International Development and the European Union, which funded the Reintegration Assistance and Development in Afghanistan programme, continued to fund such approaches until the 2021 regime change.^205,206^

#### Psychological interventions

Our search returned 33 papers describing psychological interventions with Afghans, 24 of which assessed trial effectiveness or structured evaluations (Table 3).

**Table 3:** Psychological interventions with Afghan populations

Intervention (references)	Samples	Description	Evaluation/research design	Primary outcome measures	Results
Focusing^195,202–204^	In-country Afghans; Kabul University students; Afghans in Pakistan; NGO and health worker staff	Mindfulness techniques, designed to address psychosocial issues, resilience and the culture of emotions, in culturally grounded and gender-sensitive ways	Qualitative interviews with key informants and focus groups discussion	Not applicable	Attitudes of teachers who received the psychosocial training had changed substantially: teaching styles were more relaxed, patient, helpful and stimulating; teachers involved students through role-playing, asking better-informed questions and focusing on students' emotional needs
Basic counselling training^207^	Female survivors of gender-based violence in an NGO centre in Kabul	Group counselling using our key strategies: psychoeducation, stress management, teaching of new social skills and developing new supportive networks	Open-ended, semi structured interviews with participants	Not applicable	Over 90% of the participants described improvements in social life or general health
Value-based counselling^208^	Northern Afghanistan; Afghans in Germany	Short-term psychodynamic counselling to improve self-efficacy, sense of coherence and well-being	RCT comparing value-based counselling with pharmacological treatment, at 3-month follow-up	HSCL; MINI; PHQ-9; PTSD Checklist for DSM-5; PSS-10; GAD-7; PHQ-15	At 3-month follow-up, the intervention group showed clinically meaningful reductions in depression, anxiety, PTSD symptoms, perceived stress and somatic complaints
Multi-modal day treatment ^209,210^	Afghan refugees in a specialised trauma clinic in The Netherlands	Intensive group treatment in day clinic to reduce psychological distress	Seven-year follow-up evaluation of PTSD, anxiety and depression	HSCL-25; HTQ	Mean levels of psychopathology (PTSD, anxiety, depression and psychoticism) decreased in the treatment group, but not the control group, and were sustained for up to 5 years
‘Writing for Recovery’^211,212^	Bereaved Afghan adolescents in schools in Iran; in-country Hazara girls	Group intervention aimed at reducing traumatic grief symptoms of bereaved children and adolescents through two daily 15-min writing sessions over three consecutive days	RCT with wait-list control condition	TGIC; CRIES	Writing about traumatic experiences alleviated grief symptoms; the intervention group had lower PTSD symptom severity post-intervention and at 3-month follow-up
Culturally adapted CBT (CA-CBT)^127,213,214^	Afghan male refugees in Germany; in Iran; and female refugees in Malaysia	Culturally adapted CBT	Pilot trials	GHQ-28; RHS-15	Improvements in distress and quality of life
Trauma-focused CBT (TF-CBT)^215–217^	Afghan adolescents in Iran; in-country Afghans	Memory specificity training; modified written exposure therapy	RCT with inactive control, evaluating depression and PTSD symptoms	AMT; CRIES; (Persian) MFQ	The memory training group had lower levels of depression and PTSD symptoms, maintained for up to 12 weeks follow-up
Multi-modal psychosocial intervention^218^	In-country Afghan children at risk of substance misuse	Motivational interview techniques, skill-building and art-based therapy	Qualitative evaluation	CRIES; SCARED; ASCL; SRQ-20; QOL	Significant improvement observed across all measures
‘Strong Families’^219–222^	In-country Afghan mothers and children; Afghans in Serbia; Afghans in Iran; Afghan women in Iran	Programme to improve parenting skills, child well-being and family mental health	Qualitative evaluation and RCT evaluating child mental health symptoms and parental practices and skills		
Quality-of-life therapy^223^	Kabul University female students	Psychoeducation programme on principles and skills to identify hopes and values	Quasi-experimental design evaluating subjective well-being and life satisfaction	RSWQ	General well-being of participants improved at post-test and was maintained at follow-up
‘Thinking Healthy’^224^	Afghan women who gave birth in the past year and had depressive symptoms, in Parwan province	Psychological intervention for perinatal depression based on cognitive–behavioural approaches, offered through an infant feeding scheme	Feasibility study, without a control group	PHQ-9	65% of women who attended all six intervention sessions showed decreased depressive symptoms
Adapted version of Problem Management Plus (aPM+)^225,226^	Afghans in Austria	Transdiagnostic, low-intensity psychological treatment to build skills in managing stress, problems and anger; improving self-efficacy; strengthening social support	Pilot RCT evaluation of general health, distress, symptoms of complex PTSD, self-identified problems, quality of life and integration relative to controls at 1 week follow-up		
Skills-Training of Affect Regulation – A Culture-sensitive Approach (STARC)^227^	Young Afghan male refugees in Germany	Transdiagnostic, manual-based group treatment for refugees aiming to improve emotional understanding, clarity and expression on the one hand, and adaptive emotion regulation on the other hand	Pilot RCT evaluation of improved emotion regulation and transdiagnostic symptom severity compared with a wait-list control group	DERS; GHQ-28	Decrease in emotion regulation difficulties and psychopathology, relative to waitlisted group, with treatment effects maintained 3 months post-intervention

NGO, non-governmental organisation; RCT, randomised controlled trial; HSCL, Hopkins Symptom Checklist; MINI, Mini-International Neuropsychiatric Interview; PHQ-9, Patient Health Questionnaire-9; PTSD, post-traumatic stress disorder; PSS-10, Perceived Stress Scale; GAD-7, Generalised Anxiety Disorder-7; PHQ-15, Patient Health Questionnaire-15; HSCL-25, Hopkins Symptom Checklist-25; HTQ, Harvard Trauma Questionnaire; TGIC, Traumatic Grief Inventory for Children; CRIES, Child Revised Impact of Event Scale; CBT, cognitive–behavioural therapy; CA-CBT, culturally adapted cognitive–behavioural therapy; GHQ-28, General Health Questionnaire; RHS-15, Refugee Health Screener-15; TF-CBT, trauma-focused cognitive-behavioural therapy; AMT, Autobiographical Memory Test; MFQ, Mood and Feelings Questionnaire; SCARED, Self-Report for Childhood Anxiety Related Emotional Disorders; ASCL, Afghan Symptom Checklist; SRQ-20, Self-Reporting Questionnaire-20; QOL, quality of life (ad hoc scale by researchers); RSWQ, Ryff Subjective Wellbeing Questionnaire; DERS, Difficulties in Emotion Regulation Scale.

##### Value-based counselling

Within Afghanistan, the first randomised controlled trial of a psychological intervention compared a pharmacological treatment and value-based counselling (VBC), in a study conducted by Ayoughi et al among women in Northern Afghanistan.^208^ VBC is a short-term psychodynamic intervention that aims to improve the sense of coherence and self-efficacy of clients in the course of a non-directive but carefully structured conversation.^228^ At 3-month follow-up, VBC clients reported clinically significant reductions in depression and anxiety symptoms, as well as fewer psychosocial stressors (e.g. family conflicts) and better coping responses. By contrast, symptom severity, number of stressors and coping mechanisms did not improve in patients receiving pharmacological treatment. Among Afghans in Germany, VBC clients showed a greater reduction of psychological symptoms, including depression, PTSD, perceived stress, anxiety, somatic complaints and daily functionality impairment, at post-test assessment relative to controls.^229^ VBC was integral to the first training curriculum for psychosocial counsellors in Afghanistan's healthcare system.^230^

##### Cognitive–behavioural therapy

A number of studies have evaluated culturally adapted versions of cognitive–behavioural therapy (CBT). For example, among war-bereaved Afghan adolescents in Iran, those in the experimental ‘Writing for Recovery’ group reported significant reductions in traumatic grief symptoms compared with controls;^211^ long-term effects were not assessed. Similarly, a study with Hazara adolescents found that both a written exposure therapy (five sessions) and trauma-focused CBT significantly reduced PTSD symptoms, compared with controls.^212^ Among bereaved Afghan adolescents in Iran, memory-specificity training reduced depression and PTSD symptoms at 12-week follow-up relative to controls.^215,216^ Culturally adapted CBT plus problem management (CA-CBT+), a resilience-focused intervention that utilises psychoeducation, problem-solving training, meditation and stretching exercises, was also shown to reduce psychological distress among 23 Afghan refugees in Germany at 1 year after intervention.^127,213^

The WHO has been instrumental in developing a number of scalable psychological interventions implemented with Afghans. For example, problem management plus (PM+) is a low-intensity, transdiagnostic psychological treatment aiming to enhance self-efficacy, social support and skills pertinent to coping with stress. It was adapted for Afghan refugees in Austria,^225^ and found to be effective in terms of reducing distress and PTSD symptoms and improving quality of life.^226^ It was also adapted for Afghans living in refugee camp settings in Greece,^231^ with ongoing programme evaluation.^232^ Another WHO-endorsed intervention, Thinking Healthy, was implemented among rural Afghan women with postpartum depression: a feasibility study showed strong reduction of depressive symptoms for women who completed all sessions. However, managing expectations and treatment adherence proved a major challenge, as over half of the women did not return after the first session.^224^ Lastly, a new transdiagnostic intervention, Skills-Training of Affect Regulation – A Culture-sensitive Approach (STARC), was shown to significantly improve self-reported difficulties in emotion regulation, transdiagnostic symptom severity and post-traumatic stress symptoms among young male refugees in Germany.^227^

##### Multimodal interventions

In one study, a multi-modal day-treatment programme was evaluated with survivors of torture among Afghan refugees in The Netherlands.^209^ Although causality cannot be inferred given the quasi-experimental design, symptoms were alleviated until 5 years after treatment, after which symptoms worsened but remained under baseline.^210^ Another evaluation study focused on motivational interviewing techniques, contingency management, skill-building education and art therapy techniques to prevent children at risk for substance use from declining into addictive behaviour; it showed promising results in a naturalistic study with children (*N* = 783) in Afghanistan.^218^

In turn, a retrospective study evaluated a psychosocial programme for survivors of gender-based violence in Afghanistan; it showed that 90% (*n* = 109) of women participating in the counselling groups described an improvement in their general health and social life.^207^ Another study evaluated the ‘Psychosocial Health Programme’ conducted in Kabul and Mazar-al-Sharif, with a representative sample (*N* = 296) of female survivors of gender-based violence: it found that participants reported increased self-esteem, self-efficacy and resilience, although the majority still had high symptom levels of PSTD and depression.^233^

##### Life skills training

‘Strong Families’, a programme designed to improve parenting skills as well as child well-being and family mental health, has shown promise in different contexts. For example, a pilot test in Afghanistan with 67 female caregivers and their children demonstrated clinically significant improvement in child mental health measures, along with improving parenting practices and family adjustment skills.^219^ The programme's feasibility, acceptability and effectiveness in improving child outcomes and parenting practices was also revealed for Afghan refugee families in Serbia^220^ and Afghans in Iran.^221^ Based on qualitative data, El-Khani et al note that positive changes in participants were driven by improved caregiver–child communication and reduced harshness of parenting practices. This supports a theory of change whereby the change in parenting skills is a key mechanism for reducing and preventing future child behavioural and emotional problems.^220^ Two other examples of life skills training are noteworthy. One was undertaken with 60 Afghan refugee women in Iran, showing improved social functioning and reduced mental health symptoms.^222^ Another engaged 40 female students at Kabul University, showing that quality-of-life training, as a therapeutic approach, improved subjective well-being and life satisfaction.^223^

## Discussion

This article consolidates available evidence pertaining to the mental health of conflict-affected Afghans, with a view to provide useful resources for researchers, practitioners and policy makers working in the field of MHPSS. The human, social and economic toll that violence and forced displacement have taken on the mental health of the Afghan people is evident, both in-country and in refugee settings. Clearly, risks of mental health issues are disproportionately high for women, ethnic minorities, children and youth, people with disabilities and people using drugs. Within Afghanistan, needs are so dire that the United Nations warned that the deteriorating situation in the country and the limited access to services may drive a mental health crisis with long-term and unpredictable consequences.^12^

Based on our findings, we make four recommendations to address the mental health needs of Afghans in ways that can help promote equity and foster sustainable systems of care. First, interventions need to be culturally relevant, fitting the lived experience of Afghans and their conceptualisations of mental health and well-being. This requires intensive consultation, close engagement with local Afghan stakeholders and co-ownership of services. Sustainable programmes will be those that reflect cultural features that Afghans recognise as important, such as faith, perseverance and family relationships.^196,234^ It is important, for instance, to address the physical manifestations and social embeddedness of psychosocial distress, rather than medicalise common mental health symptoms. It is also important to structure interventions able to address gender disparities within the cultural logic of Afghan worldviews, and the social stressors that affect the lives of in-country and refugee Afghans. Specifically, people in Afghanistan face ongoing everyday stressors that are inextricably rooted in economic hardships, family conflicts and restrictive social policies that drain hope and aspirations. For their part, refugees, despite often showing remarkable agency and entrepreneurship in response to adversity, face the challenges of economic participation and social inclusion in host societies, with lives often marred by cultural bereavement, loss of social status, intergenerational conflicts and changes in gender roles.

Second, vigorous investment is needed to strengthen community-based psychosocial support and evidence-based psychological interventions. Fair and equitable access to psychosocial services must remain an important goal, with specific attention to the poor, women, ethnic minorities, and children and adolescents who are the next generation of Afghans. Because many mental health problems are engendered by family-related conflicts, many Afghans might be unable or reluctant to seek professional help, given resistance from family members who act as gatekeepers to healthcare access. It is thus essential to build upon approaches that sustain well-being, strengthen capacities of individuals and families to manage stress and effectively support each other, and can be facilitated at community-level without pathologising mental health issues. Many of such interventions have been shown to be feasibly implemented and yield important benefits. More research and evaluation work will be needed to build the evidence base for psychological interventions that are adapted to Afghan languages and contexts. This includes effectiveness research, detailing what works for whom, as well as implementation research, providing knowledge about how psychosocial interventions can be scaled up. Such work is best rooted in community-based participatory approaches that embrace local voices, to establish which MHPSS interventions ‘make sense’ and are feasible in the current context. Additionally, options need to be explored how mental health interventions can be integrated within broader initiatives targeting poverty alleviation, social cohesion, peacebuilding and reconciliation.

Third, core mental health services need to be maintained at a logical point of access, such as primary health centres and general hospitals, within Afghanistan. Urgent financial support is needed to avert the implosion of Afghanistan's health system, and mental health needs must not be overlooked. A minimally acceptable level of clinical psychiatric services, such as carefully described in key documents by the Ministry of Public Health,^159,162^ must be maintained within Afghanistan's overstretched and underfunded health system. This must be paired with initiatives to reduce barriers to healthcare access and to destigmatise mental illnesses through psychoeducation. The need to prevent and mitigate drug use, especially among at-risk youth, remains a matter of urgency, especially because HIV/AIDS is on the rise in Afghanistan and misconceptions about its spread are pervasive.

Finally, humanitarian efforts must invest in building sustainable systems of care, in which different approaches (community-based psychosocial work, psychotherapeutic interventions and clinical mental healthcare) connect and reinforce each other.^235^ Within Afghanistan, the remaining MHPSS professionals need to be engaged in capacity-building and supervisory roles. In countries hosting refugees and asylum seekers, the focus of service development must include the reinforcement of social support mechanisms and access to mental health services and community-based programmes; for example, by engaging Afghans themselves in service provision.

We recognise, with considerable concern, that the task of sustaining the legacy of MHPSS work is daunting within the current socio-political context of Afghanistan. There is no clear path or easy answer as to how the legacy of MHPSS work can be extended under the current Taliban rule. There are many challenges ahead; however, past initiatives overcame many more challenges, pertaining to political insecurity, shortages of health facilities and personnel, and equitable access to quality mental healthcare. Our recommendations stem from evidence synthesised in this thematic review. One important limitation of this review is that materials were not evaluated for quality or risk of bias; however, we were able to trace both peer-reviewed and grey literature sources, include multidisciplinary work from researchers and service providers, and encompass 40 years of research and practice.

This review of past achievements points to four ways of addressing the MHPSS needs of Afghans: building cultural relevance; investing in community-based psychosocial interventions and evidence-based psychological interventions; maintaining core mental health services at logical points of access and building integrated, sustainable systems of care. Addressing the needs of vulnerable groups will remain at the forefront of MHPSS efforts. Over time, Afghans have shown a remarkable capacity for innovative solutions in the context of adversity. We believe they will continue to do so, provided the international community, donor agencies, advocacy groups, academic institutions and other stakeholders continue investing resources into promoting and protecting the mental health and psychosocial well-being of the Afghan people.

## Data Availability

Data availability is not applicable to this article as no new data were created or analysed in this study.

## Footnotes

1 Personal communication, Dr Zalmai Shinwari, HealthNet TPO, May 2022.

2 Personal communication, Dr Abdul Qawi Alimi, WHO Mental Health Consultant, June 2022.

3 Personal communication, Mariam Ahmadi, Head of the Department of Counseling at Kabul University, July 2022.

4 Personal communication, Dr Khesraw Parwiz, former Ministry of Public Health Mental Health Project Coordinator, June 2022.

5 Data from the Department of Mental Health of the Ministry of Public Health, obtained on May 2022, through Dr Majeed Siddiqi, from HealthNet TPO, June 2022.

6 Personal communication, Dr Mohammad Raza Stanikzai, United Nations Office on Drugs and Crime, June 2022.
